# Effects of eHealth-Based Multiple Health Behavior Change Interventions on Physical Activity, Healthy Diet, and Weight in People With Noncommunicable Diseases: Systematic Review and Meta-analysis

**DOI:** 10.2196/23786

**Published:** 2021-02-22

**Authors:** Yanping Duan, Borui Shang, Wei Liang, Gaohui Du, Min Yang, Ryan E Rhodes

**Affiliations:** 1 Department of Sport, Physical Education and Health Hong Kong Baptist University Hong Kong China; 2 Department of Social Sciences Hebei Sport University Shijiazhuang China; 3 Department of Health Science Wuhan Sports University Wuhan China; 4 School of Exercise Science, Physical and Health Education University of Victoria Victoria, BC Canada

**Keywords:** systematic review, meta-analysis, noncommunicable disease, multiple health behavior change, weight-related, physical activity, healthy diet, eHealth

## Abstract

**Background:**

Noncommunicable diseases (NCDs) are associated with the burden of premature deaths and huge medical costs globally. There is an increasing number of studies combining a multiple health behavior change (MHBC) intervention paradigm with eHealth approaches to jointly promote weight-related health behaviors among people with NCD; yet, a comprehensive summary of these studies is lacking.

**Objective:**

This review aims to meta-analyze the effectiveness and systematically summarize the characteristics of the relevant intervention studies for improving the outcomes of physical activity, healthy diet, and weight among people with NCD.

**Methods:**

Following PRISMA guidelines, 4 electronic databases (PsycINFO, PubMed, Scopus, SPORTDiscus) were systematically searched to identify eligible articles based on a series of inclusion and exclusion criteria. Article selection, quality assessment, and data extraction were independently performed by 2 authors. The standardized mean difference (SMD) was calculated to evaluate the effectiveness of interventions for 3 intervention outcomes (physical activity, healthy diet, and weight), and subsequent subgroup analyses were performed for gender, age, intervention duration, channel, and theory. Calculations were conducted, and figures were produced in SPSS 22 and Review Manager 5.3.

**Results:**

Of the 664 original hits generated by the systematic searches, 15 eligible studies with moderate to high quality were included. No potential publication bias was detected using statistical analyses. Studies varied in intervention channel, intensity, and content. The meta-analysis revealed that the eHealth MHBC interventions significantly promoted physical activity (SMD 0.85, 95% CI 0.23 to 1.47, *P*=.008) and healthy diet (SMD 0.78, 95% CI 0.13 to 1.43, *P*=.02), but did not contribute to a healthy weight status (SMD –0.13, 95% CI= –0.47 to 0.20, *P*=.43) among people with NCDs, compared to the control conditions. Results from subgroup analysis indicated that theory-based interventions achieved greater effect than nontheory-based interventions in promoting physical activity, and interventions with traditional approaches (SMS, telephone) were more effective than those with modern internet-based approaches in promoting healthy diet.

**Conclusions:**

The results of this review indicates that eHealth MHBC interventions achieve preliminary success in promoting physical activity and healthy diet behaviors among people with NCD. Future studies could improve the intervention design to achieve better intervention effectiveness.

**Trial Registration:**

PROSPERO International Prospective Register of Systematic Reviews CRD42019118629; https://www.crd.york.ac.uk/prospero/display_record.php?RecordID=118629

## Introduction

Noncommunicable diseases (NCDs) pose a major threat to global public health. NCDs, such as cardiovascular diseases, cancers, and diabetes, are the leading causes of death worldwide, causing 41 million deaths each year, equivalent to 71% of all deaths globally [1]. Furthermore, NCD-related medical costs significantly contribute to health care expenditure in many areas around the world [2,3].

For people with NCDs, in addition to obtaining traditional medical treatment, it is essential to adopt a healthy lifestyle through health care intervention in order to avoid further progression and relapse of NCDs [4]. In the recent 15 years, multiple health behavior change (MHBC, namely addressing no less than 2 health behaviors within a limited time period) has demonstrated early success in facilitating a healthy lifestyle among people with NCDs [5-7]. A statement in the Lancet pointed out that weight-related healthy behaviors including regular physical activity (PA) and healthy diet are promising interventions to control the NCD crisis globally [8].

The rationale of applying MHBC to weight-related behavior change is that most of the weight-related unhealthy behaviors (ie, physical inactivity, unhealthy diet) co-occur and are modifiable [6,9,10]. This assumption was empirically supported by a longitudinal study in which an unhealthy diet and physical inactivity strongly contributed to the onset of NCDs and a chain of negative effects including mortality and increasing health care costs [11]. Moreover, it was proposed that “the effect of a small step leading to a big leap forward” also applied to MHBC. A relatively easy health behavior change may serve as a gateway to an overall healthy lifestyle transition, as positive psychological factors such as self-efficacy and motivation can be boosted along with the initial behavior change, which in turn positively affects the subsequent one [9].

In applying MHBC among people with NCDs, a cost-effective mode is to employ up-to-date eHealth approaches by using information and communication technologies [12,13]. With the increasing number of people with NCDs, the traditional face-to-face intervention paradigm can hardly meet the needs of the population with NCDs. Thus eHealth, as an emerging delivery channel for health services and information using the internet and related technologies and media (eg, computers, smartphones), can be a potentially useful supplement for traditional interventions for the population with NCDs in aftercare family settings after discharge [14]. eHealth interventions also break the distance limitation and thus are highly recommended for their low cost, high efficiency, and easy data collection [15]. Many reviews have already shown substantial effects of employing eHealth approaches in addressing a single behavioral domain of either PA or healthy diet among people with NCDs [16,17]. For example, Haberlin et al [16] reviewed the use of eHealth to promote PA in cancer survivors and found that all the 10 included studies reported improvements in PA, with 8 of 10 studies reporting statistically significant changes.

There is an increasing number of studies combining the MHBC intervention paradigm with eHealth approaches to jointly promote weight-related health behaviors (PA and healthy diet) among people with NCDs [18,19]. However, a comprehensive summary of these relevant studies regarding the overall effects and study characteristics is still lacking. To fill this gap, this review mainly aimed to systematically summarize the characteristics of the relevant intervention studies and then pool the effect sizes from the relevant studies to accurately quantify the effects of those interventions on PA, healthy diet, and weight. Findings of this review can provide recommendations for researchers and clinicians to develop effective eHealth intervention programs to promote PA and healthy diet among people with NCDs.

## Methods

### Search Strategies

**Figure 1:**
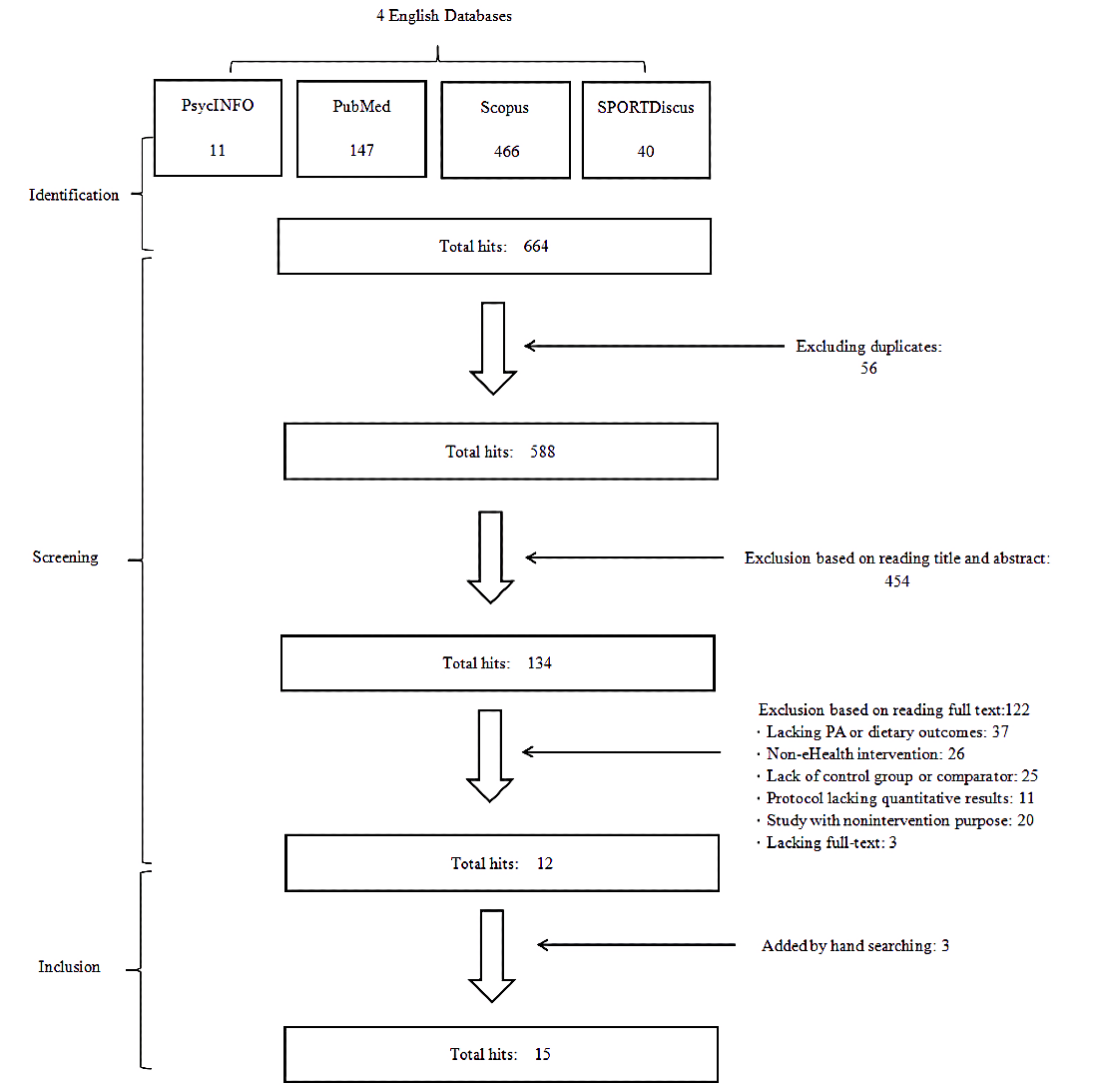
PRISMA flow chart of the search strategy and article inclusion.

This review was conducted and is reported according to the PRISMA (Preferred Reporting Items for Systematic Reviews and Meta-Analyses) guidelines, and the protocol can be retrieved from the PROSPERO database (Registration ID: CRD42019118629) [20]. According to the initial registration, we planned to search relevant articles from both English and Chinese databases. Yet, during the implementation, we found that articles retrieved from Chinese databases were unable to meet the quality standard due to misreporting of critical information (eg, participant characteristics, intervention details) [21]. Thus, we only focused on articles in English databases. A series of structured electronic searches was performed in 4 English databases including PsycINFO, PubMed, Scopus, and SPORTDiscus, focusing on MHBC eHealth interventions regarding weight-related health behaviors (PA and dietary behaviors). The procedures guiding article inclusion are presented in the flow chart in Figure 1. The specific search terms connected with Boolean operators can be seen in Multimedia Appendix 1. The searches were limited to studies with human participants and to publishing dates between 01/01/2000 and 01/03/2020.

All articles identified in the search strategy were exported into reference management software (Mendeley) for duplicate checking and further screening. The reference lists of eligible articles were further reviewed to identify other relevant studies. Relevant reviews that emerged from the search strategy were checked for any additional studies. Grey literature (eg, working papers, unpublished studies, conference proceedings or abstracts, dissertations) was not considered eligible.

### Study Inclusion and Exclusion Criteria

#### Type of Participants

The population targeted in this review was people of all genders and any age range with NCDs (eg, cardiovascular diseases, cancers, chronic respiratory diseases, diabetes). Exclusion criteria included studies with participants who were <18 years old, were pregnant or lactating, or had a special condition or other comorbidities that seriously affected their feeding ability and physical mobility (eg, physical disability).

#### Type of Intervention or Phenomena of Interest

We included studies that evaluated eHealth interventions with the primary aim of affecting behavior change that at least simultaneously incorporated PA and healthy diet behaviors. As such, studies with other irrelevant purposes (eg, investigation of behavioral patterns, investigation of the effectiveness of medical therapy, no eHealth intervention arm) were excluded.

#### Comparators

Comparators were defined as control groups without an intervention or non-eHealth intervention groups (eg, face-to-face intervention, pamphlet intervention, mass media intervention). Included studies had to compare an eHealth intervention group to at least a control group or a non-eHealth intervention group.

#### Type of Studies

Articles of randomized controlled trials were eligible for inclusion. Pure qualitative assessments of the effectiveness of an intervention were not eligible.

#### Type of Outcomes

This review primarily focused on the following behavioral outcomes measured by either subjective or objective approaches: (1) PA-related outcomes: energy expenditure, steps, time spent in moderate to vigorous PA (MVPA) and (2) healthy diet–related outcomes: energy intake, macronutrient composition (carbohydrate, protein, and lipids), core food group consumption (eg, all healthy components, vegetables, fruit, grain). The included articles should address both PA and healthy diet–related outcomes. In addition to the aforementioned primary outcomes, weight-related outcomes (eg, body weight, BMI, waist circumference, body fat, waist-to-hip ratio) were also considered as secondary outcomes.

### Data Extraction

Data extraction was performed according to a study-created extraction tool; the main framework of the extraction tool was drawn from a previously published example [22]. Two authors conducted the initial data extraction, and a third reviewer was consulted if any discrepancies in data extraction were identified. The following information was extracted: (1) basic study characteristics including the first author, date of publication, and country of origin; (2) participant characteristics including sample size, age, gender ratio (female), disease type, and recruitment location; (3) intervention characteristics including delivery channel, intervention duration and intensity, underpinning theories, control group information, and detailed intervention content; (4) outcome measures including measurement of the outcomes and measuring points; and (5) main results including intervention completion ratio and converted effect size (standardized mean difference [SMD]).

### Bias Assessments

Risk of bias was independently assessed by 2 authors according to the “Cochrane Collaboration’s tool for assessing risk of bias” (selection, performance, detection, attrition, reporting, and other biases) [23]. Disagreement between the reviewers was resolved through mutual discussion until consensus was finally reached. In addition, publication bias was assessed using a nonparametric test based on the rank correlation between the estimated treatment effect and its variance [24].

### Strategy for Data Synthesis and Meta-analysis

Results were pooled in the meta-analysis if the final values at postintervention were available. For the articles with continuous data, numbers of participants, mean scores, and SDs of the outcome variables were extracted to calculate the SMD: (m_1_-m_2_) /√[(s_1_^2^+ s_2_^2^) / 2], where m is the mean and s is the SD.

For the articles with dichotomous data, the numbers of people in each category of both intervention and control groups were extracted to calculate the odds ratio (OR): N_a_*N_d_ / N_b_*N_c_, where N_a_ is the number of adherents in the intervention group, N_b_ is the number of adherents in the control group, N_c_ is the number of nonadherents in the intervention group, and N_d_ is the number of nonadherents in the control group.

For the convenience of further calculation, the ORs were arithmetically converted to SMDs using a spreadsheet [25]. It should be noted that the effect size of some negative outcomes (eg, fat intake, BMI, unhealthy diet) were reverse coded into positive values.

For studies with multiple effect sizes in a particular scope of outcome (eg, PA-related outcomes, light PA, MVPA simultaneously), we used the weighted arithmetic averaging method to pool the effect sizes into a synthesized size [26]. Taking the study of Bantum et al [27] as an example, in this study there were 2 effect sizes regarding PA-related outcomes (SMD .16, light PA; SMD .38, MVPA). Since the sample sizes of these 2 effective sizes were equal (50% weighting each), the synthesized SMD for PA was (0.16 + 0.38)/2 = 0.27.

For the issues of missing data and statistics (eg, cases such as SDs and means not reported), we first resorted to statistical conversion (eg, convert 95% CI to SD). If it could not be statistically converted, we then contacted the authors directly for the datasets. If neither approach worked out, we excluded the unqualified studies listwise for the meta-analyses.

Finally, effect sizes were synthesized using a random effects model [28]. We adopted the random effects model because it allows inferences that generalize beyond the studies included in the specific meta-analysis [28]. A positive SMD reflects the between-group difference in favor of the eHealth MHBC intervention group over the control group (ie, increase in outcomes regarding PA, healthy diet, and healthy weight such as healthy BMI range). The pooled effect sizes are presented in forest plots that allow readers to see the information from the individual studies that went into the meta-analysis at a glance [29]. Heterogeneity was assessed using the recommended I² for Cochrane reviews [30]. Subgroup analyses were performed based on 5 binary variables: (1) gender (ratio of female participants ≥50% vs <50%), (2) age (participants’ mean age ≥55 years vs <55 years), (3) intervention duration (≥24 weeks vs <24 weeks), (4) theory (whether the intervention was guided by theory), and (5) intervention channel (pure SMS or telephone vs internet- or web-based).

All data calculations (publication bias tests, effect size syntheses, heterogeneity tests, subgroup analyses) in this meta-analysis were conducted using SPSS 22.0 [31] with the syntaxes provided by Field and Gillett [28]. All the qualitative extractions (information extraction, risk of bias assessment) and figure productions were facilitated by Review Manager 5.3 [32].

## Results

### Study Characteristics

The initial systematic search yielded a total of 664 articles (see Figure 1). After duplicate deletion and screening of abstracts and full texts, 12 articles met the inclusion criteria, with 3 additional articles added by hand searches. In total, 15 studies met the inclusion criteria and did not meet the exclusion criteria targeting improvements in PA, healthy diet, and weight status among participants with NCDs. Due to the oversize issue, the main contents of each study are summarized in Multimedia Appendix 2. Of the 15 studies identified, 4 were conducted in the United States [26,33-35], 2 each in Canada [36,37] and India [38,39], and 1 each in China [18], Pakistan [40], Korea [41], the Netherlands [19], New Zealand [42], and the United Kingdom [43]. Notably, 1 study recruited its sample globally in Canada, the United Kingdom, and the United States [44]. Study participant numbers (intervention group and control group together) ranged from 59 [41] to 683 [37], with a mean sample size of 315 participants (SD 215 participants). The participants’ average age ranged from 42.3 years [41] to 73.0 years [44]. For the gender ratio, 2 studies notably recruited predominantly or all (≥80%) male participants [38,42], while another 2 studies recruited female participants (>80%) [26,41]; 1 study did not report gender information [39]. Various NCD types were covered, among which, the top 3 addressed diseases were heart disease [18,34,37,42,43], cancer [19,26,41,44], and diabetes [33,38-40].

Various intervention channels and media were applied, including web sites or pages, telephone counselling, and SMS. Compared with the traditional SMS-only and telephone-only interventions, adding web-based materials in interventions were more prevalent (8/15, 53%). Notably, many studies adopted a mixed-channel intervention, such as combining a web-based intervention with SMS reminders and offline peer support or group meetings [26,33] or combining web-based intervention material with SMS or telephone reminders [18,19,41]. The intervention durations ranged from 6 weeks [26] to 1 year [39,43]. Of the included studies, 9 designed interventions of no shorter than 24 weeks, whereas 6 studies designed interventions shorter than 24 weeks. The majority (9/15, 60%) of the included interventions were designed based on a particular theory, and among the interventions with a theoretical backdrop, the Transtheoretical Model (6/15, 40%) and Social Cognitive Theory (4/15, 27%) were the top 2 frequently supporting theories.

All 15 studies evaluated both PA and healthy diet–related outcomes. However, 2 studies [34,35] did not provide SDs of both the PA and healthy diet postintervention outcomes. We failed to obtain these missing results from the corresponding authors via email, thus making these studies ineligible for the meta-analysis. In addition, 4 of the 15 studies assessed weight-related outcomes [33,38-40]. The intervention completion rate (mean numbers of participants who finished the entire intervention divided by the number of participants at baseline) ranged from 70.9% [39] to 96.7% [19].

### Bias and Heterogeneity Assessments

**Figure 2:**
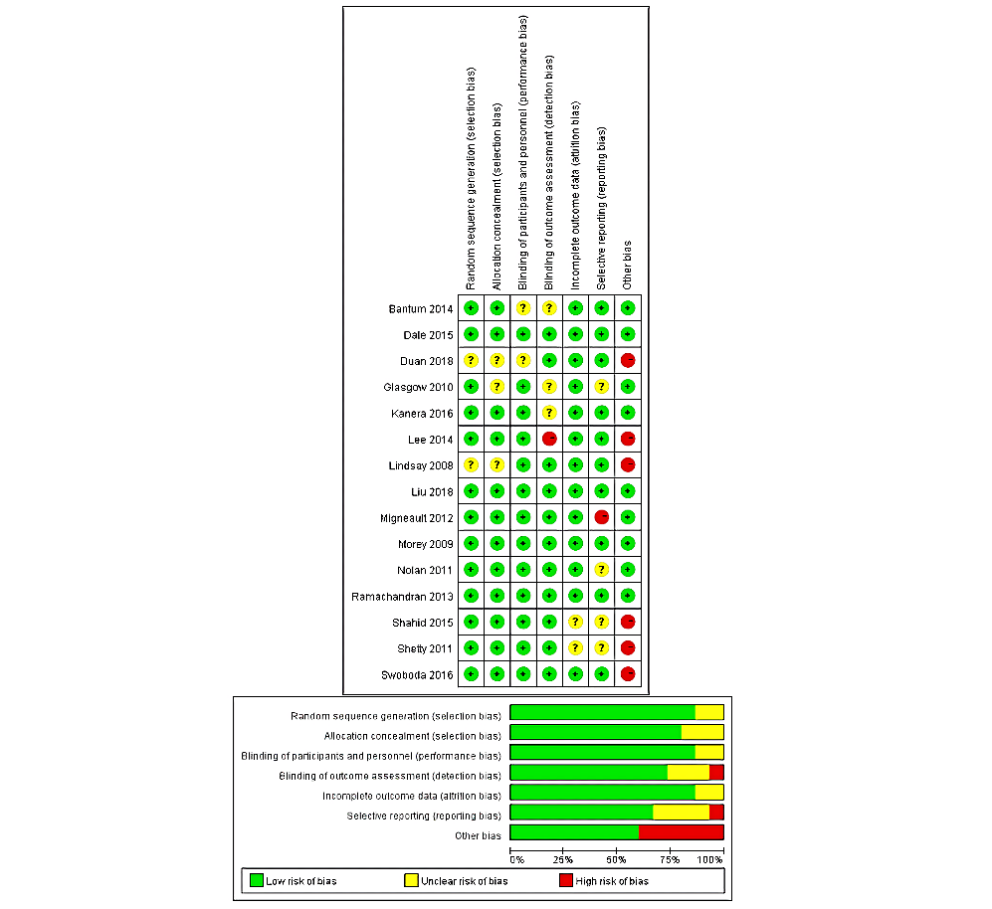
Risk of bias in individual studies (k = 15).

As can be seen in Figure 2, the risk of bias assessment indicated that the included articles were quite high in quality. For the publication bias examination, the statistical results showed no potential publication bias regarding the 3 intervention outcomes of PA (Kendall tau=.21, *P*=.33), healthy diet (Kendall tau=.26, *P*=.22), and weight (Kendall tau=.33, *P*=.50).

### Intervention Effectiveness

**Figure 3:**
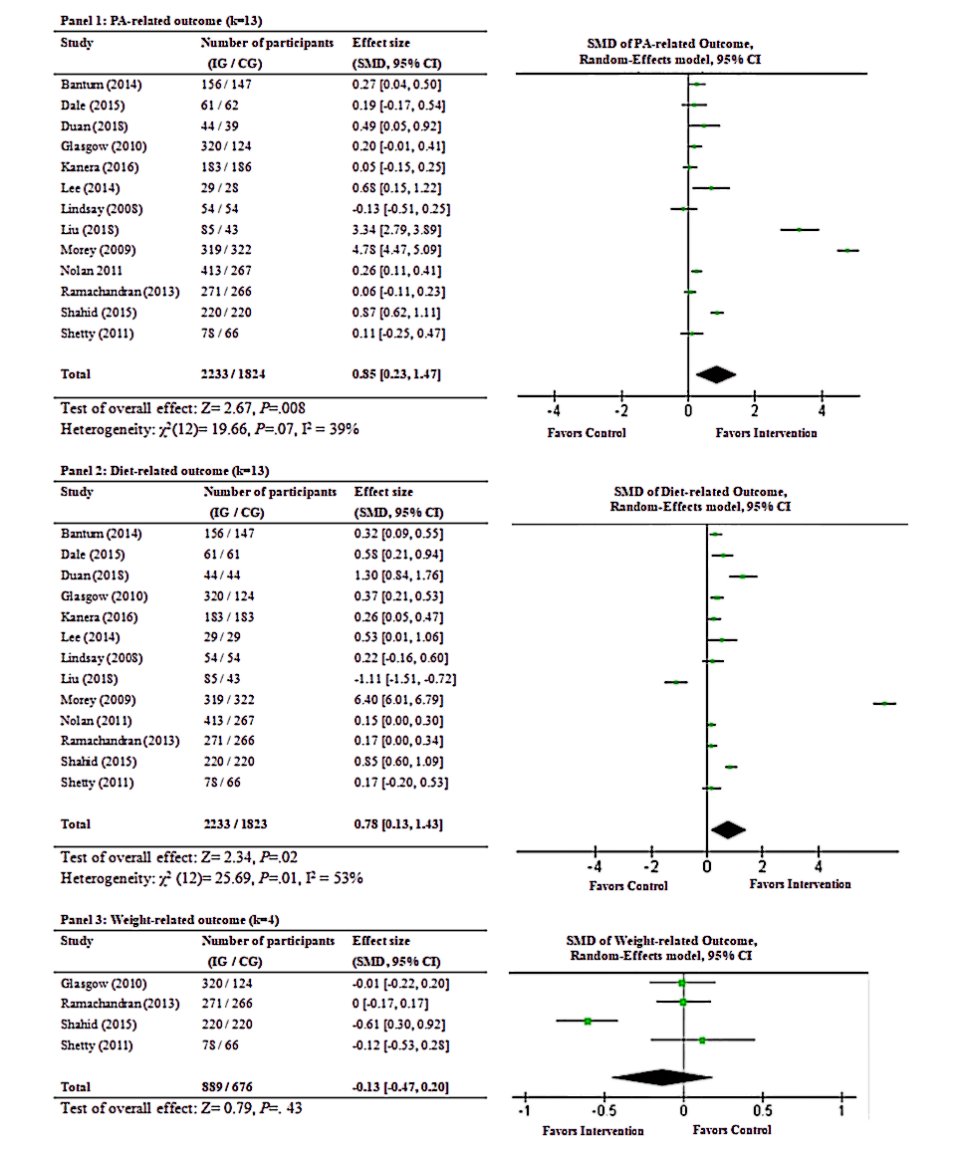
Meta-analysis of outcomes related to physical activity (PA; panel 1), diet (panel 2), and weight (panel 3). CG: control group; IG: intervention group.

In the following sections, the synthesized results regarding PA-related, diet-related, and weight-related outcomes are introduced individually. The main results are visualized in the forest plot in Figure 3.

#### PA-Related Outcomes

The synthesized effect size from the 13 studies demonstrated significant differences in total PA between intervention and control groups at postintervention (SMD 0.85, Z= 2.67, 95% CI 0.23 to 1.47, *P*=.008; see panel 1 in Figure 3). According to the rule of thumb [45], an SMD of 0.85 should be considered a large effect size. The heterogeneity test did not show significance among PA-related outcomes (χ^2^_12_=19.66, *P*=.07, I^2^=39%). For the subgroup analyses, gender (χ^2^_1_=0.44, *P*=.51), age (χ^2^_1_=1.78, *P*=.18), intervention duration (χ^2^_1_=0.98, *P*=.32), and intervention channel (χ^2^_1_=0.75, *P*=.39) were all not significantly related to the intervention effectiveness. Noticeably, whether the intervention was based on theory (χ^2^_1_=2.41, *P*=.12) marginally approached the subgroup significant difference. Specifically, interventions based on theories (SMD 1.22, Z=2.13, 95% CI 0.10 to 2.34, *P*=.03) achieved better effectiveness than nontheory-based interventions (SMD 0.30, Z=1.83, 95% CI –0.02 to 0.61, *P*=.07).

#### Healthy Diet–Related Outcomes

In terms of the remaining 13 studies, significant differences in healthy diet behaviors were demonstrated between intervention and control groups at postintervention (SMD 0.78, Z=2.34, 95% CI 0.13 to 1.43, *P*=.02; see panel 2 in Figure 3). According to the rule of thumb [45], an SMD of 0.78 should be considered a medium effect size. Concerning the heterogeneity tests, significant heterogeneity was revealed among healthy diet–related outcomes (χ^2^_12_=25.69, *P*=.01, I^2^=53%). For the subgroup analyses, gender χ^2^_1_=2.19, *P*=.14), age (χ^2^_1_=.46, *P*=.50), intervention duration (χ^2^_1_=.21, *P*=.65), and whether the intervention was based on theory (χ^2^_1_=1.28, *P*=.26) were not significantly related to the intervention effectiveness. However, the intervention channel was marginally related to the intervention effectiveness (χ^2^_1_=2.59, *P*=.10). Specifically, interventions with traditional SMS or telephone counselling (SMD 1.54, Z=2.05, 95% CI 0.07 to 3.01, *P*=.04) achieved better effectiveness than web-based interventions (SMD 0.30, Z=1.70, 95% CI –0.05 to 0.64, *P*=.09).

#### Weight-Related Outcomes

There were only 4 studies examining weight-related outcomes. Results did not show a significant difference in weight status between intervention and control groups at postintervention (SMD –0.13, Z=0.79, 95% CI –0.47 to 0.20, *P*=.43; see panel 3 in Figure 3). Insignificant heterogeneity was shown among weight-related outcomes (χ^2^_3_=2.82, *P*=.42). I^2^ could not be calculated due to the small number of studies. Since subgroup analyses is not recommended when any subgroup has <4 studies [46], we did not conduct the subgroup analysis for the weight-related outcomes.

## Discussion

### Principal Findings

This review systematically identified 15 studies that investigated the effectiveness of MHBC eHealth interventions aimed at improving PA-, healthy diet–, and weight-related outcomes among people with NCDs. The results showed that the MHBC eHealth interventions significantly promoted both PA and healthy diet outcomes among people with NCDs. However, the results did not show significant intervention effectiveness regarding weight changes. These results are in partial agreement with the review findings of Amireault et al [47], which showed MHBC interventions regarding PA- and healthy diet–related behaviors could significantly improve PA behaviors among cancer survivors. Yet, the findings from our review differ from the findings of the review by Alageel et al [48], which demonstrated the majority of MHBC interventions for patients with cardiovascular diseases could not achieve significant changes in either PA behavior or fruit and vegetable consumption. Regarding effect sizes, MHBC eHealth interventions achieved a large effect size in terms of PA (SMD 0.85) and healthy diet (SMD 0.78) behaviors [45]. The effect sizes from our meta-analysis are larger than the effect sizes of 2 meta-analyses synthesizing eHealth interventions on PA behavior in older people (SMD 0.79) [49] and fruit and vegetable intake in a healthy population (SMD 0.26) [50]. This might be due to the fact that we targeted people with NCDs who are already impacted by diseases. Thus, the populations in our meta-analyses might be more motivated and willing than other healthy populations to change their health behaviors, thus reaping more positive intervention effects [51].

Though all the included intervention studies adopted an MHBC eHealth approach, there was still a high variability in participants (eg, cultural backgrounds, age), intervention characteristics (eg, intervention channel, content, duration), and outcome measurements. This indicates that, as an emerging intervention paradigm, MHBC eHealth interventions are in the exploratory phase and do not have relatively well-acknowledged intervention guidelines or standards such as CONSORT [52].

Despite this high variability, we still found notable trends. First, the eHealth intervention channel has clearly changed from traditional SMS-based or telephone counselling to modern, multimedia, web-based or smartphone-based interventions. Since 2015, except for 1 article using a traditional telephone-based intervention [34], all interventions were web-based. With the convenience of an internet connection and the popularity of smartphone usage in daily life, it has been predicted that future interventions via smartphone apps are promising for better promotion of a healthy lifestyle among people with NCDs [13].

Second, the intervention contents of most included studies mainly focused on health behavior education and counselling without substantial behavioral tutorials; this might be due to the present limitations of the eHealth intervention channel. The commonly used intervention paradigm was to select a particular health behavior change theory as the framework and further promote the effective elements (e.g., motivation, planning, and self-regulation) of the chosen theory. Such commonly used approaches did achieve significant medium-sized effectiveness of the intervention (SMD around 0.8) in changing the weight-related health behaviors of PA and healthy diet. To further increase the intervention effectiveness, a dual-process approach (ie, focusing on both conscious and nonconscious processes of behavior change) [53,54] and a social-ecological approach (ie, involving policy-level, environmental, and personal factors) may be prudent [55,56].

Third, regarding the outcome measurements, the majority of studies used self-report measures. Self-report is a feasible, economic, and time-saving approach for data collection [57], but it also has limitations of high subjectivity and low accuracy caused by social desirability and reporting bias. With the advancement of technology, more objective approaches of data collection regarding PA (eg, geo-information, system-based recording; wearable device–based recording), healthy diet (eg, food photography, computer-assisted recall), and weight (eg, electronic scale data collection) are recommended to improve the accuracy during data collection [58].

Fourth, follow-up analyses were generally lacking in the intervention designs. Most of the included studies measured outcomes twice (preintervention baseline and postintervention). Long-term and maintenance effects of MHBC eHealth interventions on both PA and healthy diet behaviors among people with NCDs were not therefore validated. Given that the MHBC eHealth intervention is in its infancy, these first studies were mainly to examine whether eHealth interventions with PA and healthy diet could be effective. As advocated [6,13], the next stage in this research is to explore how and under what conditions these initial changes can be maintained by adding longer follow-up designs.

Regarding the intervention effectiveness, we adopted analytical methods proposed in previous relevant systematic review articles to pool the effect sizes by different outcomes [47,59]. This approach can effectively reduce the difficulty of data processing and improve the clarity of results presentation. On the other hand, this approach somewhat ignored the overall effect of a particular intervention program. Our results only indicated that MHBC eHealth interventions targeting people with NCDs, on average, can significantly promote PA and healthy diet; it has yet to be confirmed that MHBC eHealth interventions can successfully promote an entire pattern of weight-related health behaviors among people with NCDs. In the future, as the volume of MHBC studies increases, a well-acknowledged index to indicate the overall effect size of MHBC interventions is expected to be developed for better evaluation of intervention effects.

Interestingly, the post hoc subgroup analyses did not find any significant moderators influencing intervention effectiveness. However, the results did reveal some potential moderators (ie, whether the intervention was based on theory, intervention channel) reaching marginal significance, which is noteworthy given the small sample of studies. Interventions based on theories achieved significant effectiveness for promoting PA behaviors, while those without any theoretical basis did not. The results are consistent with previous meta-analyses regarding using theory in health behavior promotion [60-63]. And interestingly, interventions with traditional intervention media achieved better effectiveness than web-based interventions for promoting healthy diet behaviors. The reason might be that some of the included web-based interventions were lacking direct virtual communication or periodic reminders, which might potentially hinder the intervention effectiveness [36,43]. This inference could be supported by previous research that web-based interventions can achieve a desirable effect only when adding additional methods of communication with participants, especially the use of SMS or text messages [61].

Also interestingly, intervention duration did not have a significant impact on intervention effectiveness for either PA or healthy diet behaviors; a possible explanation might be that the intervention frequency and intervention sequence were not considered [64]. In summary, we recommend future MHBC eHealth interventions combine web-based intervention material with traditional periodic calling or reminders through virtual contact and pay more attention to intervention quality and sequence arrangement among multiple health behaviors for achieving better intervention effectiveness.

### Limitations

Three limitations of this review should be acknowledged. First, despite our best efforts to conduct a thorough literature search in the limited databases, it may still have resulted in the omission of suitable topics or related studies due to not including key terms or research outside the time span that was searched. Second, bias might be present in some included studies because they lacked a registered protocol, used inappropriate statistical methods, and had missing information [39,40,43], which therefore suggests additional caution should be taken in interpreting the findings from these trials and the pooled effect sizes. Third, there was a high degree of heterogeneity (ie, participant characteristics, intervention types and lengths, and outcome measurements), and the small number of studies could lead to cautious interpretation of the synthesized results.

### Conclusions

To the best of our knowledge, this review is the only study that has attempted to synthesize the literature regarding the effectiveness of MHBC eHealth interventions on PA, healthy diet, and weight for people with NCDs. Such MHBC eHealth studies have emerged in recent years as a new trend in aftercare rehabilitation settings. The current review significantly contributes to the eHealth- and NCD-related literature by identifying research priorities and providing preliminary evidence for clinical decision making. This review indicates that MHBC eHealth interventions have obtained preliminary success in promoting PA and healthy diet behaviors among people with NCDs. The identification of critical intervention characteristics such as being theory-based and adding communication elements to web-based intervention material are essential for maximizing the effects of MHBC eHealth interventions in promoting weight-related behaviors among people with NCDs. Based on this review study, it is expected that further investigations will make recommended improvements on the intervention design in order to ultimately enhance the well-being of people with NCDs.

## Appendix

Multimedia Appendix 1 The search strategies of the systematic review.

Multimedia Appendix 2 Detailed records of eligible studies (k= 15).

## Abbreviations

MHBC: multiple health behavior change
MVPA: moderate to vigorous physical activity
NCD: noncommunicable disease
OR: odds ratio
PA: physical activity
PRISMA: Preferred Reporting Items for Systematic Reviews and Meta-Analyses
SMD: standardized mean difference
