# Supplementary material for: Effects of eHealth-Based Multiple Health Behavior Change Interventions on Physical Activity, Healthy Diet, and Weight in People With Noncommunicable Diseases: Systematic Review and Meta-analysis
Source: J Med Internet Res. 2021 Feb 22;23(2):e23786. doi: 10.2196/23786 (PMC8074786; doi:10.2196/23786)
Supplement: Multimedia Appendix 1 [file jmir_v23i2e23786_app1.docx]

**Multimedia Appendix 1.** The search strategies of the systematic review.

4 English databases: Totally 664 articles were identified (by the date 01-03-2020)

- Scopus 466
- SPORTDiscus 40
- PubMed 147
- PsycINFO 11
- Scopus: 466 results

Searching algorithm:

( ALL ( ehealth  OR  mhealth  OR  "mobile app*"  OR  telemedicine  OR  telehealth ) )  AND  ( ALL ( mhbc  OR  "multiple health behavi*"  OR  "multiple risk behavi*"  OR  "multiple risk factor*" ) )  AND  ( ( ALL ( obes*  OR  *weight OR  "body mass index"  OR  bmi  OR  "energy balance" ) )  OR  ( ( ALL ( exercise  OR  "motor activity"  OR  "physical activity" ) )  AND  ( ALL ( nutrition  OR  feeding  OR  food  OR  diet*  OR  intake  OR  consumption ) ) ) )  AND ( EXCLUDE ( PUBYEAR ,  1999 )  OR  EXCLUDE ( PUBYEAR ,  1998 ) )  AND  ( LIMIT-TO ( LANGUAGE ,  "English" ) )

- SPORTDiscus: 40 results

Searching algorithm:

((((TX exercise) OR (TX "motor activity") OR (TX "physical activity")) AND ((TX nutrition) OR (TX feeding) OR (TX food) OR (TX diet*) OR (TX intake) OR (TX consumption))) AND (((TX obes*) OR (TX *weight) OR (TX "body mass index") OR (TX BMI) OR (TX "energy balance")) OR (((TX exercise) OR (TX "motor activity") OR (TX "physical activity")) AND ((TX nutrition) OR (TX feeding) OR (TX food) OR (TX diet*) OR (TX intake) OR (TX consumption))))) AND (((TX MHBC) OR (TX "multiple health behavi*") OR (TX "multiple risk behavi*") OR (TX "multiple risk factor*")) AND ((((TX exercise) OR (TX "motor activity") OR (TX "physical activity")) AND ((TX nutrition) OR (TX feeding) OR (TX food) OR (TX diet*) OR (TX intake) OR (TX consumption))) AND (((TX obes*) OR (TX *weight) OR (TX "body mass index") OR (TX BMI) OR (TX "energy balance")) OR (((TX exercise) OR (TX "motor activity") OR (TX "physical activity")) AND ((TX nutrition) OR (TX feeding) OR (TX food) OR (TX diet*) OR (TX intake) OR (TX consumption))))))

- Pubmed: 147 results

Searching algorithm

 ((((mhbc) OR "multiple health behavi*") OR "multiple risk behavi*") OR "multiple risk factor*" AND (("2000/01/01"[PDat] : "2018/12/31"[PDat]))) AND (((((ehealth) OR mhealth) OR "mobile app*") OR telemedicine) OR telehealth AND (("2000/01/01"[PDat] : "2018/12/31"[PDat]))) AND (((((obes*) OR weight) OR "body mass index") OR "energy balance" AND (("2000/01/01"[PDat] : "2018/12/31"[PDat]))) OR ((((exercise) OR "motor activity") OR "physical activity" AND (("2000/01/01"[PDat] : "2018/12/31"[PDat]))) AND ((((((nutrition) OR feeding) OR food) OR diet*) OR intake) OR consumption AND (("2000/01/01"[PDat] : "2018/12/31"[PDat]))) AND (("2000/01/01"[PDat] : "2018/12/31"[PDat]))) AND (("2000/01/01"[PDat] : "2018/12/31"[PDat]))) AND (("2000/01/01"[PDat] : "2018/12/31"[PDat]))

- PsycINFO 11 results

Searching algorithm

[((((nutrition) OR (feeding) OR (food) OR (diet*) OR (intake) OR (consumption)) AND ((exercise) OR ("motor activity") OR ("physical activity"))) OR ((obes*) OR (weight) OR ("body mass index") OR (BMI) OR ("energy balance"))) AND ((MHBC) OR ("multiple health behavi*") OR ("multiple risk behavi*") OR ("multiple risk factor*")) AND ((ehealth) OR (mhealth) OR (mobile app*) OR (telemedicine) OR (telehealth))](https://search-proquest-com.lib-ezproxy.hkbu.edu.hk/myresearch/savedsearches.checkdbssearchlink:rerunsearch/1491029/SavedSearches?site=psycinfo&t:ac=SavedSearches)
