# Supplementary material for: Effects of eHealth-Based Multiple Health Behavior Change Interventions on Physical Activity, Healthy Diet, and Weight in People With Noncommunicable Diseases: Systematic Review and Meta-analysis
Source: J Med Internet Res. 2021 Feb 22;23(2):e23786. doi: 10.2196/23786 (PMC8074786; doi:10.2196/23786)
Supplement: Multimedia Appendix 2 [file jmir_v23i2e23786_app2.doc]

**Multimedia Appendix 2. Detailed records of eligible studies (k= 15).**

| **First author (year)**  **Nation** | **Participant characteristics** | **Intervention characteristics** | **Outcome Measures** | **Main Results** |
| --- | --- | --- | --- | --- |
|  | **Sample size, Age [M(SD)],**  **Female ratio, NCD type, recruitment location** | **Channel, Theory, Duration, Intensity, Control group, and Intervention contents** | **Measuring points, PA and diet measurement** | **Completion ratio, results of PA, diet, and BMI (all converted to SMD)** |
| Bantum (2014)  US | N= 352 (IG=176;CG=176);  Age: 43.5 (6.9)  Female ratio: 46%  NCD type: Cancer  Recruitment location: oncology clinics | Channel:Web-based with SMS remind and peer facilitator support;  Non-theory based;  Duration and intensity: 6-week, each week 30-35 web pages;  Control group: wait-list control group  Intervention contents: A self-management online workshop targeting skills building, information about specific content, and the encouragement of weekly action plans to build self-efficacy. Contents include improving diet by making healthier food choices, increasing exercise, stress management via relaxation training, improving communication with health care providers, processing and communicating emotional experiences to people inside of one’s existing social network, goal and action plan setting, feedback offering. | 2 measuring points, baseline and 6-month  PA: Self-report by using Godin Exercise Questionnaire (min/week)  Diet: Self-report by using Block Food Frequency Questionnaire (number of servings of FVI) | Intervention completion ratio: 88.6%  PA: SMD= 0.27 [0.04, 0.50]  Diet: SMD= 0.32 [0.09, 0.55] |
| Dale (2015)  New Zealand | N= 123(IG=61; CG=62)  Age: 59.5 (11.1)  Female ratio: 18.7%  NCD type: CHD  Recruitment location: in-hospital,before discharge  from hospital after their cardiac event | Channel:SMS with supporting website and pedometer;  SCT theory based;  Duration and intensity: 24-week; 6 messages/week, Bi-weekly tips and guidance on the supporting website.  Control group: usual care control  Intervention contents: Contents including smoking cessation, limiting alcohol consumption, FVI intake and maintaining regular PA. Messages were tailored to participants’ name and preferred time of day to receive messages. Bidirectional messaging allows participants to report their progress on the behavior change. The supporting website offered biweekly tips from the research team via a participant blog, graphs displaying their pedometer step counts, and short video messages from role models and medical professionals. | 2 measuring points, baseline, 24-week (end of all data collection).  Binary score of PA and Diet  PA: (1 indicates ≥14 units of MVPA/week; 0 indicates ≤13 units of MVPA/week) as measured by the Godin Leisure Time Physical Activity Questionnaire.  Diet: FVI (1 indicates ≥5 servings daily; 0 indicates ≤4 servings daily) from the New Zealand Health Survey. | Intervention completion ratio: 93.4%  PA: SMD= 0.19 [-0.17, 0.54]  Diet: SMD= 0.58 [0.21, 0.94] |
| Duan (2018)  China | N= 114 (IG=60; CG=54)  Age: 48.5 (13.3)  Female ratio: 53.5%  NCD type: CHD  Recruitment location: outpatients from cardiac rehabilitation center | Channel:Web-based + short massage reminder;  HAPA theory-based;  Duration and intensity: 8-week; intervention intensity dependent on participants’ voluntary visits.  Control group: waiting control group  Intervention contents: week 1 and week 5—risk perception, outcome expectancies, and goal setting; week 2 and week 6—development of action plans; week 3 and week 7—revision and adjustment of previous action plans and development of coping plans; and week 4 and week 8—revision and adjustment of previous coping plans and development of behavior-specific social support. selected behavior change techniques were addressed in the intervention to facilitate the implementation and maintenance of behavior. Moreover, examples of role models were provided throughout the intervention to support patients to set goals, develop plans, and increase their self-efficacy. | 2 measuring points, baseline and 8-week  Measured by subjective self-report  PA: by short version of the "IPAQ" (adherence; 0=not, 1=yes)  Diet: Measured by 7-day self report recall (servings of FVI, adherence; 0=not; 1=yes) | Intervention completion ratio: 72.8%  PA: SMD= 0.49 [0.05, 0.92]  Diet: SMD= 1.30 [0.84, 1.76] |
| Glasgow (2010)  US | N= 463 (IG=331; CG=132)  Age: 58.4 (9.2)  Female ratio: 49.8%  NCD type: Diabetes and Obesity  Recruitment location: family setting | Channel:Web-based + periodic promoting call + group meeting;  Social ecological model+ self-management model;  Duration and intensity: 4-month; intervention intensity dependent on participants’ voluntary visits.  Control group:enhanced usual care  Intervention contents: Participants were asked to select initial easily achievable goals in 3 domains of medication adherence, exercise, and food choices. Daily progress can be recorded, weekly feedback can also be provided accordingly. The website also offer graphical display of the patient’s blood pressure, cholesterol level, PA and diet results; a moderated forum; and community resources (e.g., healthful recipes, printable handouts) for healthy lifestyles, as well as features to enhance user engagement, such as rotating quiz questions and motivational tips; action plan building and revising; problem-solving and barrier overcoming strategies. Participants also received periodic prompting using a computer-based telephone system that initiated outbound calls, received inbound calls, provided motivational information, and collected data. | 2 measuring points, baseline and 4-month  Measured by subjective self-report  PA: by Community Health Activities Model Program for Seniors (CHAMPS) Questionnaire (Kilo-Cal/week)  Diet: Eating habits were measured by subjective self-report by “Starting The Conversation” scale (total points) | Intervention completion ratio: 79.2%  PA: SMD= 0.20 [-0.01, 0.41]  Diet: SMD= 0.37 [0.21, 0.53]  BMI: SMD= -0.01 [-0.22, 0.20] |
| Kanera (2016)  Netherlands | N= 462 (IG=231; CG=231)  Age: 55.9 (11.4)  Female ratio: 79.9%  NCD type: Cancer  Recruitment location: cancer registries hospitals | Channel:Web-based + email reminder;  Integrated Model for Change and SCT;  Duration and intensity: 6-month; weekly delivery intervention intensity dependent on participants’ voluntary visits.  Control group:usual-care control group  Intervention contents: The intervention is tailored to personal characteristics of cancer survivors. 7 out of the total 8 modules are self-management modules target the specific needs associated with the relevant topic of healthy lifestyle including PA and diet. Principles of Problem Solving Therapy and behavior change techniques such as conscious raising, identifying pros and cons, identifying barriers and providing solutions, persuasive communication, self-monitoring, social modeling, goal setting, action, and coping planning were applied to encourage self-management. Participants were made specifically aware of their own behavior in relation to the norms. Detailed and personalized feed-back targeting attitudes, social support, self-efficacy, barriers, and intentions toward behavior change was provided. Text, photos, videos of fellow survivors and specialists, and hyperlinks to other sources of information were used for this purpose. | 2 measuring points, baseline and 6-month  PA: Measured by SQUASH (min/week)  Diet: Measured by Dutch Standard Questionnaire on Food intake (Vegetable: grams/day; Fruit:servings/day) | Intervention completion ratio: 96.7%  PA: SMD= 0.05 [-0.15, 0.25]  Diet: SMD= 0.26 [0.05, 0.47] |
| Lee (2014)  Korea | N= 59 (IG=30; CG=29)  Age: 42.3 (5.7)  Female ratio: 100%  NCD type: Cancer  Recruitment location: cancer registries in hospitals | Channel:Web-based + SMS reminder;  TTM theory based;  Duration and intensity: 12-week; bi-weekly delivery (twice a week).  Control group:attention control group  Intervention contents: including 4 parts: assessment, education (tailored information provision), action planning (goal setting, scheduling, diary keeping and automatic  feedback. Specifically, The educational content were as follows; enhancing exercise and dietary behaviour in cancer survivors; the importance of normal weight management; barriers to sustainability of exercise and diet behaviour; considerations when planning exercise and diet; a consequences such as QOL and survival of the regular exercise and a balanced diet; and exercise and dietary guidelines for cancer survivors. The data on actual behaviours and what were recommended were compared visually on the Web screen. This information was used to give daily feedback on goal achievement as part of the automatic feedback  Portion. | 2 measuring points, baseline and 12-week  PA: Measured by 7-day exercise diary (exercise adherence: 0=not; 1=yes)  Diet: Measured by 3-day dietary recall (FVI adherence: 0=not; 1= yes) | Intervention completion ratio: 96.6%  PA: SMD= 0.68 [0.15, 1.22]  Diet: SMD= 0.53 [0.01, 1.06] |
| Lindsay (2008)  UK | N= 108 (IG=54; CG=54)  Age: 62.9 (N/A)  Female ratio: 32.4%  NCD type: CHD  Recruitment location: coronary heart disease registries in hospitals | Channel:Web-based;  not theory-based;  Duration and intensity: 6-month; voluntarily log on the website portal.  Control group:control group  Intervention contents: The participants in the intervention group could interact in one of five dedicated closed groups, with facilitation from researchers. Other detailed information was unknown. | 2 measuring points, baseline and 6-month  PA: by asking ‘how many days during a typical week you spend in moderate exercise’ where a higher score indicates more time spent in exercise (active days)  Diet: was measured by adding a series of variables  together, which are standardized from the Health Survey for England (total score ranging from 6–30) | Intervention completion ratio: N/A  PA: SMD= -0.13 [-0.51, 0.25]  Diet: SMD= 0.22 [-0.16, 0.60] |
| Liu (2018)  Canada | N= 128 (IG=85; CG=43)  Age: 56.9 (0.8)  Female ratio: 47.7%  NCD type: Hypertension  Recruitment location: family settings | Channel:Web-based;  TTM theory-based;  Duration and intensity: 4-month; weekly updated, voluntarily log on the website portal.  Control group: control group  Intervention contents: including e-counseling enabled the participants to set their  own goals or to select the interventions used to reach their behavioral goal. The user-driven e-counseling group received weekly e-mails that enabled participants to select their areas of lifestyle change using text and video web links embedded in the  e-mail. The web links contained information about the following: constructing exercise and diet plans setting behavioral goals based on their readiness, self-monitoring of lifestyle behaviors, and blood pressure, resolving ambivalence for change, increasing efficacy for initiating change, and reviewing social and cognitive behavior skills for relapse prevention to maintain adherence. | 2 measuring points, baseline and 4-month  PA: Objectively measured by pedometer (steps/day)  Diet: Self-report by using Block Food Frequency Questionnaire (number of servings of FVI) | Intervention completion ratio: 89.4%  PA: SMD= 3.34 [2.79, 3.89]  Diet: SMD= -1.11 [-1.51, -0.72] |
| Migneault (2012)  US | N= 337(IG=169; CG=168)  Age: 56.5 (11.0)  Female ratio: 70.0%  NCD type: Hypertension  Recruitment location: urban safety-net hospital and from  four affiliated community health centers | Channel:Computer-based telephone counselling;  TTM & SCT based;  Duration and intensity:32-week; 1 counselling by call / week.  Control group: usual care control  Intervention contents: Besides SCT and TTM, the intervention also incorporated principles of motivational interviewing, and was tailored to the user’s values. 32 calls: The first 3 calls introduced the three targeted behaviors and their role in BP control, and described the system. Subsequent calls were arranged as modules on medication adherence (8 calls), physical activity (12 calls), and diet (9 calls), and were delivered in the order chosen by the participant. Each call consisted of (a) an introduction, (b) a section for reporting health information collected on study-issued home measurement devices, and (c) theory-based interactive education and counseling on the targeted behavior. | 4 measuring points,  Baseline, 4-month, 8-month, 12-month  PA: by interviewer-administered 7-day physical activity recall (1.weekly MVPA minutes;2.total daily energy expenditure; reaching or not 150 min MVPA)  Diet: by Food Frequency Questionnaire (FFQ) (Diet quality score 0-100, higher better) | Intervention completion ratio: 74.0%  PA: N/A  Diet: N/A |
| Morey (2009)  Canada, UK and US | N= 641 (IG=319; CG=322)  Age: 73.0 (5.0)  Female ratio: 53.9%  NCD type: cancer  Recruitment location: North Carolina Central Cancer Registry | Channel:telephone counselling + mailed print material  Based on SCT+TTM  Duration and intensity:1 year, 15 sessions and 8 prompts.  Control group:delayed intervention control group  Intervention contents: Participants received a personalized workbook. The introductory pages featured bar graphs comparing participants’ current lifestyle behaviors and weight status with recommended levels. These pages also recommended 15 minutes of strength training exercise every other day; 30 minutes of endurance exercise each  day; consumption of at least 7 servings (for women) or 9 servings (for men). of fruits and vegetables per day restriction of saturated fat to less than 10% of energy intake; and a 10% weight loss goal during the 12-month study period. Workbook chapters provided standardized content on exercise and a healthy calorie-restricted diet. Participants also received a pedometer, exercise bands (3 levels of resistance), an exercise poster depicting 6 lower extremity strength exercises, a table guide to food portioning (Portion Doctor, Portion Health Products, St Augustine Beach, Florida), and personalized record logs to self-monitor daily exercise and dietary intake (including a fat gram booklet to assist with self-monitoring).  Each participant was assigned a health counselor for the 12-month period. Counseling sessions were conducted weekly during the first 3 weeks, every other week for 1 month, and then monthly. Each telephone session was 15 to 30 minutes in duration and served to enhance social support and self-efficacy. During each telephone call the counselor worked with the participant to monitor progress, provide reinforcement, explore strategies in overcoming barriers, field questions, direct participants to appropriate resources, and establish future goals. Automated telephone messages by the study principal investigator provided additional, intermittent reinforcement. Every 12 weeks, participants received a tailored 2-page progress report newsletter with a motivational greeting, a graph depicting behavioral change in the target behaviors related to the goals, and a sign-off message that was tailored to the participant’s readiness to change behavior. | 2 measuring points, baseline and 1-year  PA: Physical activity was assessed by the Community Health Activities Model Program for Seniors questionnaire (minutes/week)  Diet: Dietary intake data were averaged from 2 unannounced 24-hour recalls at baseline and at 12 months using the interactive Nutrition Data System  for Research software (FVI servings + saturated fat intake grams). | Intervention completion ratio: 84.3%  PA: SMD= 4.78 [4.47, 5.09]  Diet: SMD= 6.40 [6.01, 6.79] |
| Nolan (2011)  Canada | N= 683 (IG=415; CG=268)  Age: 59.0 (0.5)  Female ratio: 49.8%  NCD type: CHD  Recruitment location: family setting | Channel:Telephone-based;  non theory-based (only with TTM’s stage);  Duration and intensity:6-week; 6 weekly 1-hour group teleconference with group members and experts.  Control group: active control group  Intervention contents: The telephone based intervention is a form of group-based application of motivational interview.Subjects identified their priority for lifestyle change (diet, exercise, or smoking) and they were taught to selfassess their stage of readiness for change. Motivational interviewing guidelines were used to focus group discussion on (1) salient lifestyle goals identified by subjects, (2) progress in resolving ambivalence about lifestyle change, and (3) experiences of increased efficacy in initiating or maintaining change. Quality control of this intervention was maintained by weekly supervision by teleconference. | 2 measuring points, baseline and 4-month  PA: Adherence to the PA guideline (1=yes, 0=no) by subjective report  Diet: Adherence to the diet guideline (1=yes, 0=no) by subjective report. | Intervention completion ratio: 74.7%  PA: SMD= 0.26 [0.11, 0.41]  Diet: SMD= 0.15 [0.00, 0.30] |
| Ramachandran (2013)  India | N= 537 (IG=271; CG=266)  Age: 46.0 (4.7)  Female ratio: 0%  NCD type: Diabetes  Recruitment location: workplace | Channel:SMS-based;  TTM based;  Duration and intensity:6-month; 2-4 messages per week.  Control group:standard care control group  Intervention contents: Participants in the intervention group received mobile phone messages at frequent intervals. These messages contained information about healthy lifestyle, the benefits of PA and diet, cues to start PA and healthy dietary practices, and strategies to avoid relapse and remain motivated to maintain PA and healthy dietary habits. Each SMS contained fewer than 160 characters and 60–80 messages were created and sent by the set order according to TTM. The assumption was that the participants would move from a pre-action stage to an action stage. The timing  and frequency of mobile phone messaging were tailored to the participants’ preferences. | 5 measuring points baseline, after treatment (6-month), 12-month, 18-month, 24-month  PA: Measured by adherence to the PA guideline (adherent (>=2 days/week following guideline) , non-adherent) by subjective report  Diet: Measured by adherence to the diet guideline (adherent non-adherent) by subjective report. | Intervention completion ratio: 90.4%  PA: SMD= 0.06 [-0.11, 0.23]  Diet: SMD= 0.17 [0.00, 0.34]  BMI: SMD= 0 [-0.17, 0.17] |
| Shahid (2015)  Pakistan | N= 440 (IG=220; CG=220)  Age: 49.0 (8.8)  Female ratio: 38.6%  NCD type: diabetes  Recruitment location: outpatient services in hospital | Channel:mobile phone counselling;  non-theory-based;  Duration and intensity:4 months, every 15 days a call.  .Control group:control group  Intervention contents:participants in intervention arm were directly called after every 15 days for a period of 4 months (8 calls in total) regarding intake of medications, physical activity and healthy eating. Both the groups were given SMBG form to monitor their blood glucose levels. Participants were trained by the diabetes educationist in the correct use of the glucometer and to correctly write the readings in the form. The written information leaflets related to information regarding diet given by the nutritionist, initiating and maintaining healthy lifestyles, symptoms of hypoglycemia and hyperglycemia and complications of DM were provided to both the groups on the visit of randomization. | 2 measuring points, baseline and 4-month  PA: adherence to recommended PA level (direct asking by phone, detailed measuring method not mentioned)  Diet: adherence to healthy diet habit (direct asking by phone, detailed measuring method not mentioned) | Intervention completion ratio: not mentioned  PA: SMD= 0.87 [0.62, 1.11]  Diet: SMD= 0.85 [0.60, 1.09]  BMI: SMD= -0.61 [-0.80, -0.42] |
| Shetty (2011)  India | N= 215 (IG=110; CG=105)  Age: 50.1 (9.9)  Female ratio: not mentioned  NCD type: diabetes  Recruitment location: not mentioned | Channel:SMS-based;  non-theory-based;  Duration and intensity:1 year, receive SMS once in 3 days.  Control group:standard care control  Intervention contents: The intervention group received SMS once in 3 days as a reminder to strictly follow the regimen of dietary modification, physical activity and drug schedules. Totally 102 messages, written in English consisted of varied instructions on medical nutrition therapy (MNT), physical activity, reminders,on following drug prescription and messages also on healthy living habits. All patients had attended patient education programme, during the initial and follow up visits to the clinic. | 2 measuring points, baseline and 1-year  PA: adherence to recommended PA level (measured by questionnaire developed by the authors)  Diet: adherence to healthy diet habit (measured by questionnaire developed by the authors) | Intervention completion ratio: 70.9%  PA: SMD= 0.11 [-0.25, 0.47]  Diet: SMD= 0.17 [-0.20, 0.53]  BMI: SMD= 0.12 [-0.21, 0.45] |
| Swoboda (2016)  US | N= 60 (IG=41; CG=19)  Age: 56.1 (6.7)  Female ratio: 68.5%  NCD type: CVD  Recruitment location: community organizations (e.g., libraries, senior centers) and workplaces | Channel:Telephone-based;  non-theory-based (goal-setting and reaching + motivational interview);  Duration and intensity:16-week; bi-weekly phone call (totally 7 calls made).  Control group: attention control group  Intervention contents: Participants in the intervention group received 1 face-to-face coaching session with a registered dietitian trained in motivational interviewing and decision support coaching to encourage changes in diet and PA tailored to CVD risk factors. Self-set goals pertaining to both diet and PA were established at baseline and at each of 7 subsequent biweekly phone calls, and action plans were created to help participants implement their goals. This pragmatic intervention did not assign specific goals; instead, motivational interviewing and decision coaching were used to support participants to make personalized diet and activity goals. During telephone calls, the participant and dietitian discussed their success with self-set goals and created new goals following goal attainment. If participants did not meet their goals, decision support coaching was used to clarify preferences, and problem solving occurred for participant-identified barriers to goal attainment. Descriptions of goals and action plans were recorded and emailed to the participant after each coaching session. | 2 measuring points, baseline and 16-week  PA: by subjective self-report by long version of the "IPAQ" (MET-min/week)  Diet: by Food Frequency Questionnaire (servings/day) | Intervention completion ratio: 90.2%  PA: N/A  Diet: N/A |

*Note.* C (in superscript): Published in Chinese language; IG: Intervention group; CG: Control group; M: Mean; SD: Standard deviation; PA: Physical activity; FVI: Fruit and Vegetable Intake; CHD: Coronary heart disease; CVD: Cardiovascular disease; SMS: Short message service; SCT: Social Cognitive Theory; N/A: Not available; MHD: Maintenance hemodialysis; HAPA: Health Action Process Approach; TTM: Transtheoretical Model; IPAQ: International Physical Activity Questionnaire; SQUASH: Short Questionnaire to Assess Health Enhancing Physical Activity; MVPA: Moderate-to-vigorous physical activity; OR: Odds ratio; QOL: Quality of life; SMD: standard mean difference; We-chat: a mega-popular communication app in smart phone among Chinese; Weibo: The Chinese version of Twitter. Intervention completion ratio: means number of participants finished the entire intervention divided by number of participants at baseline.
